# Investigation of adhesion status of *Candida* species to the surface of resin materials produced at different angles with additive manufacturing

**DOI:** 10.1186/s12903-024-04505-1

**Published:** 2024-06-27

**Authors:** Omer F. Turanoglu, Esra Talay Cevlik, Caner Vural

**Affiliations:** 1 Department of Prosthodontics, Faculty of Dentistry, Aydın Adnan Menderes University, Aydın, Efeler, 09100 Turkey; 2 Department of Biology, Molecular Biology Section, Faculty of Science, Pamukkale University, Denizli, Pamukkale, 20160 Turkey

**Keywords:** 3D printing, Surgical guide, Microbial adhesion, *Candida*

## Abstract

**Background:**

The aim of this study was to evaluate the adhesion of *Candida glabrata*, *Candida albicans*, *Candida krusei*, *Candida parapsilosis* and *Candida tropicalis* yeasts to disk-shaped resin materials produced from resin which used in the production of surgical guide with 0, 45 and 90-degrees printing orientations by Liquid Crystal Display additive manufacturing technology.

**Methods:**

Disk-shaped specimens were printed with surgical guide resin using the Liquid Crystal Display production technique in 3 printing orientations (0, 45 and 90-degrees). Surface roughness and contact angle values were evaluated. Real-Time PCR analysis was performed to evaluate *Candida* adhesion (*C. glabrata*, *C. albicans*, *C. krusei*, *C. parapsilosis* and *C. tropicalis*) Field emission scanning electron microscope (FESEM) images of the materials were obtained.

**Results:**

Specimens oriented at 45-degrees demonstrated higher surface roughness (*P* < .05) and lower contact angle values than other groups. No significant difference was found in the adhesion of *C. glabrata*, *C. albicans*, and *C. parapsilosis* among specimens printed at 0, 45, and 90-degrees orientations (*P* > .05). A higher proportion of *C. krusei* and *C. tropicalis* was found in the specimens printed at orientation degrees of 45 = 90 < 0 with statistical significance. Analyzing the adhesion of all *Candida* species reveals no statistical disparity among the printing orientations.

**Conclusions:**

The surface roughness, contact angle, and adhesion of certain *Candida* species are affected by printing orientations. Hence, careful consideration of the printing orientation is crucial for fabricating products with desirable properties. In 45-degree production, roughness increases due to the layered production forming steps, whereas in 0-degree production, certain *Candida* species exhibit high adhesion due to the formation of porous structures. Consequently, considering these factors, it is advisable to opt for production at 90-degrees, while also considering other anticipated characteristics.

## Background

Treatment predictability is increased by digitally positioning implants before surgery. In this context, surgical guides produced by additive manufacturing (AM) are frequently used in implant placement today [1, 2]. In AM, the printing orientation plays a pivotal role in determining the direction of layering and the configuration of layers, thereby shaping the geometry of the surface. Given that surface geometry significantly influences surface properties, the choice of printing orientation for denture bases holds substantial importance in shaping the surface properties of three-dimensional (3D) printed dentures. Key material properties impacted by the printing orientation include surface properties, color stability, cleanability of the denture base, and adhesion of microorganisms [3]. However, there are few studies regarding the relationship between surface properties that change with the printing orientation and the adhesion properties of microorganisms in the literature [4, 5].

It has been recognized that *Candida* is implicated in the development of lesions such as candidiasis and angular cheilitis [6]. Moreover, it is closely associated with premalignant and malignant oral lesions [7]. Notably, leukoplakia lesions infected with *Candida* present a heightened risk of malignancy [8]. *Candida* infections have been identified to play a contributory role in the development of oral epithelial dysplasia and neoplasia. An association has been observed between oral epithelial dysplasia and members of the genus *Candida* present in the oral cavity, with species such as *C. albicans, C. tropicalis, C. glabrata, C. parapsilosis*, and *C. krusei* being commonly encountered [9–11]. While *Candida* species are generally recognized as opportunistic pathogens capable of causing various oral infections [12], the precise relationship between *Candida* infections and oral cancer remains uncertain [13]. Further research is needed to elucidate whether *Candida* infections directly contribute to the development of oral cancer.

Conventional methods for identifying microorganisms, such as cultivation on agar plates, have long been used. However, there is an urgent need for rapid and reliable methods to identify microorganisms in routine clinical diagnostics. To meet this need, various chromogenic media and biochemical reaction-based test kits are currently used. However, clinical specimens (saliva, biopsy, dental plaque) may contain microorganisms belonging to different subspecies or clades, posing challenges in obtaining definitive results for timely decision-making. Molecular biology methods offer greater accuracy in such scenarios. In particular, Real-Time PCR is a frequently preferred, rapid, and reliable method widely used to identify microorganisms [14, 15]. Additionally, studies which the Real-Time PCR technique has been utilized have described the advantages of using Real-Time PCR analysis over conventional methods for evaluating the adhesion of *Candida* species [16, 17].

The aim of this study was to investigate the adhesion of *Candida* species (*C. glabrata, C. albicans, C. krusei, C. parapsilosis*, and *C. tropicalis*) to disk-shaped samples designed and fabricated with three different printing orientations (0, 45, and 90-degrees) using the AM method. The null hypotheses of the study were:

H01 Hypothesis: Printing orientation does not affect *Candida* species adhesion.

H02 Hypothesis: There is no difference among *Candida* species when their adhesion to samples obtained by AM is evaluated.

## Methods

### Sample preparation

The disk-shaped resin samples were fabricated using the Liquid Crystal Display (LCD) 3D printing method (Ackuretta FreeShape 120, Ackuretta Technologies, Taipei, Taiwan) using resin commonly used in the production of surgical guide (MACK4D guide 405 clear/transparent Model Resin; MACK4D GmbH, Neukieritzsch, Germany). Disk-shaped materials with a diameter of 10 mm and a thickness of 2 mm, were designed according to the ISO 10993-12:2021 standard [4]. The design of the materials was created using 3D computer-aided design software (Blender v.3.5.0; Blender Foundation, Amsterdam, Netherlands), with printing orientations set at 0, 45, and 90-degrees and fabricated with at a layer thickness resolution of 100 μm. The fabricated materials were cleaned with isopropyl alcohol (%99,9 purity) for 5 min using an ultrasonic cleaning device (CLEANI, Ackuretta Technologies, Taipei, Taiwan), according to the manufacturer’s guidelines. The polymerization was then completed using a UV irradiator emitting light at a wavelength of 405 nm (UV CURIE; Ackuretta Technologies, Taipei, Taiwan), and the process lasted for 3 min.

### Surface roughness measurement

Surface roughness measurements of the prepared materials were performed using a profilometer device (Surftest SJ-210, Mitutoyo Corp., Kanagawa, Japan). The average surface roughness value (Ra) was determined by measuring (µm) three regions identified at the center of the sample surface. Measurements were made at a speed of 0.5 mm/sec, with a cut-off measurement length of λc = 0.25 [18].

### Pilot study and sample size determination

A pilot study was conducted prior to the study to determine the appropriate sample size. Adhesion data (CFU/material) obtained from the pilot study were analyzed by one-way analysis of variance using SPSS/PC Version 21.0 (SPSS Inc., Chicago, IL, USA). The mean and the common standard deviation values of the obtained data were calculated to determine the effect size for five yeast species using the G-Power program, version 3.1.9.4 (Düsseldorf, Germany). The calculated effect sizes were 3.532, 0.546, 7.053, 5.43, and 1.246 for *C. glabrata*, *C. albicans*, *C. krusei*, *C. parapsilosis*, and *C. tropicalis* respectively. The analysis was then continued using the lowest effect size value. Consequently, the sample size was determined to be 36 disk-shaped resin samples, with 12 samples in each group produced from each production angle, ensuring a power of 0.80 and a significance level of 0.05.

### Preparation of the solutions

Fusayama-Meyer artificial saliva (KCl: 0.4; NaCl: 0.4; CaCl_2_.2H_2_O: 0.906; NaH_2_PO_4_.2H_2_O: 0.690; Na_2_S.9H_2_O: 0.005; CH_4_N_2_O: 1 (g/L)), Yeast Extract Peptone (YEP) (peptone: 20; dextrose: 20; yeast extract: 10 (g/L)), and PBS solution (Na_2_HPO_4_: 1.44; KCl: 0.2; KH_2_PO_4_: 0.245; NaCl: 8 (g/L), pH 7.2) were prepared for use in *Candida* cell attachment studies. CHROMAgar^TM^ *Candida* (CHROMagar Microbiology, Paris, France) medium was purchased from a supplier. 12 samples from each material group, produced with a 3D printer at 0, 45, and 90-degrees printing orientation, were placed in 30 mL clean glass vials. PBS solution, culture media, and glass vials were autoclaved at 121 °C for 15 min.

### Microbial adhesion test

*C. albicans* ATCC 64548, *C. krusei* ATCC 6258, *C. tropicalis* RSSK 665, *C. glabrata* (clinical isolate), and *C. parapsilosis* ATCC 22019 were inoculated onto sterile YEP agar using the streak plate method. The inoculated plates were incubated at 37 °C for 24–36 h. After the incubation period, the purity of the cultured cells was assessed. Each *Candida* species was then aseptically transferred to 5 ml YEP broth tubes and incubated at 37 °C for a further 24–36 h to facilitate growth in liquid culture.

After the incubation period, 1 mL of each *Candida* species was transferred to 1.5 mL sterile microcentrifuge tubes and centrifuged at 5000 rpm for 5 minutes. After centrifugation, the supernatants were discarded, then the cell pellets were mixed with 1 mL of PBS, and the tubes were vortexed until the cells were suspended. The cell suspensions were adjusted to 0.5 (approximately 1.5 × 10^5^ CFU/mL) optical density (OD) at 540 nm using a UV-Vis spectrophotometer. A final volume of approximately 10^3^ CFU/mL of a *Candida* mixture was inoculated into each material group. The samples were then incubated in a shaking incubator at 150 rpm and 37 °C for 2 h. After incubation, materials were removed from the bottles with sterile forceps and gently immersed three times in sterile PBS to wash out the unattached cells. *Candida* cells that did not adhere to the materials were removed in this manner. Any excess fluid remaining on the material was gently absorbed with a clean napkin. Each material was placed in microcentrifuge tubes containing 1 mL of PBS and the tubes were vortexed at full speed for two minutes to detach the cells.

Detection and enumeration of adhered *Candida* cells were analyzed using a Real-Time PCR device (LightCycler^®^ 96 System, Roche Diagnostics, Germany). For this purpose, fungal genomic DNA was extracted from adherent cell solutions obtained from each sample in the vortexing step. The amount and purity of the extracted DNA samples were determined by measuring the absorbance at 260/280 nm using a microvolume spectrophotometer (Nanodrop 2000c, Thermo Scientific, USA). The FastStart Essential DNA Green Master Kit (Roche Diagnostics GmbH, Germany) was used to quantify the extracted DNA samples by Real-Time PCR [19]. The species-specific PCR primer sets were used to quantify each of the five *Candida* species in Real-Time PCR experiments (Table 1).

**Table 1:** Primer sets utilized for the quantitative assessment of *Candida* species via Real-Time PCR

Target	Primer set	Oligonucleotide sequence (5’→ 3’)	Length (bp)	Annealing temp. (°C)
*C. albicans*	Calb F	TTTATCAACTTGTCACACCAGA	273	51
	Calb R	ATCCCGCCTTACCACTACCG		
*C. glabrata*	Cgact1 F	GACGGCGATTATGAGTTAGGAG	102	53
	Cgact1 R	GTAGCATCTGTGCAGGTAGTT		
*C. krusei*	Trfp4 F	AGGCAGCAGACTTGTACCTT	183	54
	Trfp4 R	TGCCCAGTTTCGAGGTGAGA		
*C. parapsilosis*	Sadh F	ACCCGTTGTGAGAAGTGCCA	124	57
	Sadh R	ACCAAGCCTATGTCCGCAACT		
*C. tropicalis*	Trf4 F	TGTTGGTGGTCTTGGTGGGT	108	57
	Trf4 R	ACCCCCAAATTGTCTAATGCAC		

Real-Time PCR protocols were performed as follows: For *C. albicans* and *C. krusei*, an initial pre-incubation step at 95 °C for 10 min was followed by an amplification step consisting of 45 cycles at 95 °C for 10 s, 54 °C for 10 s, and 72 °C for 12 s. For *C. glabrata*, the PCR protocol included a pre-incubation step at 95 °C for 10 min, followed by amplification steps of 45 cycles at 95 °C for 10 s, 53 °C for 10 s, and 72 °C for 6 s. Similarly, for *C. parapsilosis* and *C. tropicalis*, the pre-incubation step was performed at 95 °C for 10 min, followed by amplification steps of 45 cycles at 95 °C for 10 s, 57 °C for 10 s, and 72 °C for 6 s. A melting step was then performed at 95 °C for 10 s, followed by a step at 65 °C for 60 s, and finally at 97 °C for 1 s. The procedure was completed with a cooling step at 37 °C for 30 s. The *Candida* cells adhering to the materials were calculated as copy DNA/material according to the standard curves previously established for each species.

### FESEM analysis

For surface topography analysis, three samples were selected from each working group, with one material subjected to vortexing and one left unvortexed. The selected samples were coated with an 80 − 20% gold-palladium mixture under an appropriate vacuum using a gold coating apparatus (Quorum Q150R ES, Quorum Technologies Ltd., East Grinstead, United Kingdom) for 15 min. The samples were then analyzed in a FESEM instrument (Zeiss SUPRA 40 VP, USA) [20]. The surface topography of the materials was examined along with the assessment of surface attachment of *Candida* cells.

### Contact angle measurement

The sessile drop method was employed to measure the contact angle using a goniometer device (phx300, SEO, Busan, Republic of Korea). The image of the water (4 µL) dropped onto the material was automatically measured (mean = 10) [21].

### Statistical analysis

Statistical analyses were performed using an SPSS statistical program (SPSS/PC Version 25; SPSS Inc., Chicago, IL, USA). Descriptive statistics were calculated for surface roughness and Real-Time PCR results, including mean, standard deviation, minimum, and maximum values. Normally distributed data were compared by one-way analysis of variance (ANOVA), and the Bonferroni test was used for pairwise comparisons. Welch ANOVA and post-hoc Tamhane tests were used to analyze data that did not show homogeneity of variance. All tests were performed at a significance level of 0.05. The Shapiro-Wilk test was used to determine whether the surface roughness data of the materials were normally distributed, and the Kolmogorov-Smirnov test with Lilliefors correction was used to determine whether the data obtained from the Real-Time PCR analyses were normally distributed. Levene’s test was used to determine homogeneity of variances.

## Results

The descriptive statistics and comparison of the surface roughness values are presented in Tables 2 and 3.

**Table 2:** Surface roughness values (Ra, µm) described through descriptive statistics

Printing Orientation	*n*	Mean ± SD	Minimum	Maximum
0°	12	0.6176 ± 0.1501	0.4157	0.9543
45°	12	3.4201 ± 1.3014	1.5417	5.7203
90°	12	0.7751 ± 0.1738	0.5420	1.0740

SD: Standard Deviation

**Table 3:** Surface roughness values (Ra, µm) described through descriptive statistics

Printing Orientation		Mean Difference	SE	*P*
0°	45°	-2.8024	0.3782	0.000*
	90°	-0.1575	0.0663	0.078
45°	0°	2.8024	0.3782	0.000*
	90°	2.6449	0.3790	0.000*
90°	0°	0.1575	0.0663	0.078
	45°	-2.6449	0.3790	0.000*

*P = .05, SE: Standard Error

Comparison of surface roughness values was evaluated by Welch ANOVA (F_Welch_(2,19,512) = 28,162, *P* < .05) followed by a post-hoc Tamhane test.

**Fig. 1:**
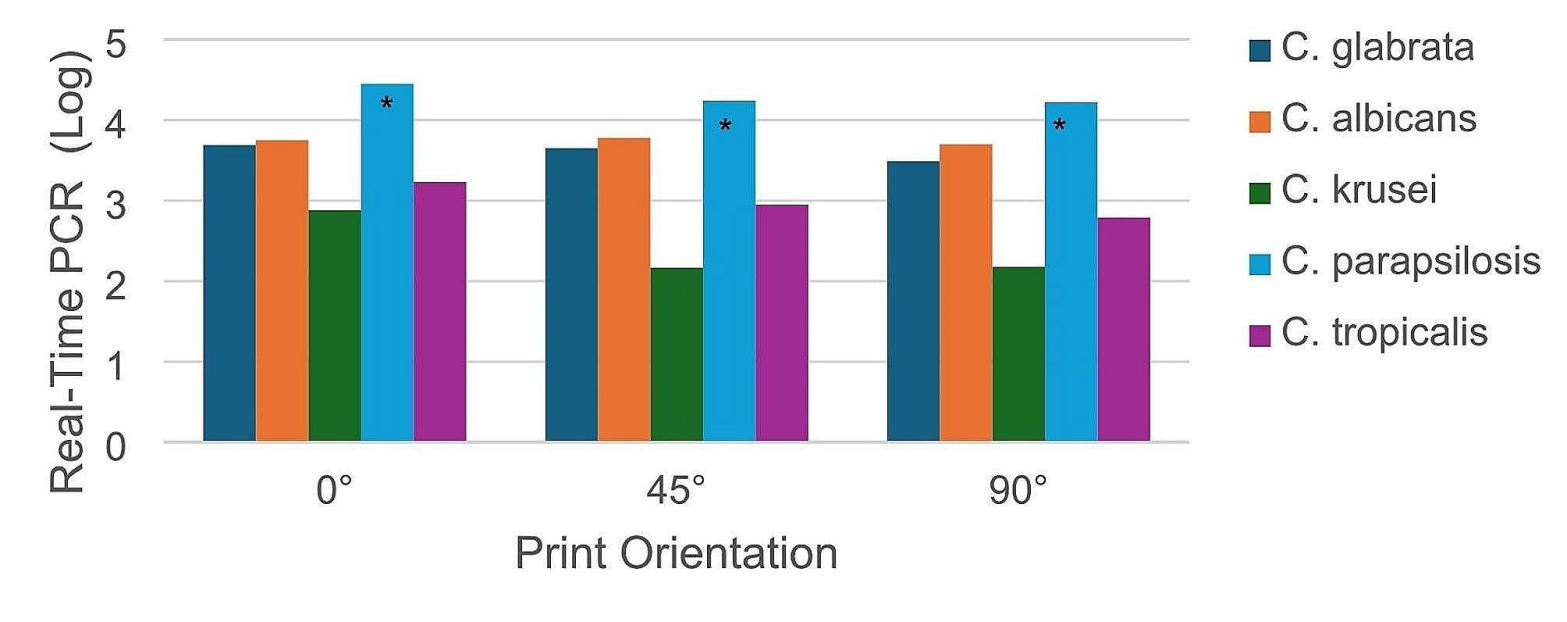
Column graph derived from Real-Time PCR findings

The descriptive statistics for the Real-Time PCR data are presented in Table 4 and the graph derived from Real Time PCR findings is given in Fig. 1.

**Table 4:** Statistical analysis summarizing Real-Time PCR results

Printing Orientation	*Candida*	*N*	Mean	SD	Minimum	Maximum
0°	*C. glabrata*	12	3.69	0.39	3.00	4.27
	*C. albicans*	12	3.75	0.27	3.21	4.24
	*C. krusei*	12	2.88	0.77	1.55	4.42
	*C. parapsilosis*	12	4.45	0.36	3.93	5.09
	*C. tropicalis*	12	3.23	0.29	2.79	3.65
	*Total*	60	3.60	0.69	1.55	5.09
45°	*C. glabrata*	12	3.65	0.23	3.30	3.96
	*C. albicans*	12	3.78	0.22	3.39	4.28
	*C. krusei*	12	2.17	0.57	1.02	2.98
	*C. parapsilosis*	12	4.24	0.28	3.96	4.82
	*C. tropicalis*	12	2.95	0.22	2.62	3.40
	*Total*	60	3.36	0.80	1.02	4.82
90°	*C. glabrata*	12	3.49	0.53	2.12	4.06
	*C. albicans*	12	3.70	0.34	2.94	4.30
	*C. krusei*	12	2.18	0.34	1.58	2.93
	*C. parapsilosis*	12	4.22	0.58	3.17	5.13
	*C. tropicalis*	12	2.79	0.29	2.24	3.33
	Total	60	3.27	0.83	1.58	5.13

SD: Standard Deviation

The amount of *Candida* species adhering to materials fabricated at a 0-degree printing orientation was evaluated using Welch ANOVA (F_Welch_ (4,28,136) = 22,118, *P* < .05), followed by a post-hoc Tamhane test for further analysis. The results are shown in Table 5.

**Table 5:** The adhesion quantity of *Candida* species on materials fabricated with a 0-degree printing orientation (Tamhane test)

*Candida*		Mean Difference	SE	*P*
*C. glabrata*	*C. albicans*	-0.06750	0.13523	1.000
	*C. krusei*	0.80480	0.24820	0.049*
	*C. parapsilosis*	-0.76133	0.15310	0.001*
	*C. tropicalis*	0.45442	0.14008	0.039*
*C. albicans*	*C. glabrata*	0.06750	0.13523	1.000
	*C. krusei*	0.87230	0.23472	0.024*
	*C. parapsilosis*	-0.69383	0.13012	0.000*
	*C. tropicalis*	0.52192	0.11451	0.002*
*C. krusei*	*C. glabrata*	-0.80480	0.24820	0.049*
	*C. albicans*	-0.87230	0.23472	0.024*
	*C. parapsilosis*	-1.56613	0.24545	0.000*
	*C. tropicalis*	-0.35038	0.23755	0.829
*C. parapsilosis*	*C. glabrata*	0.76133	0.15310	0.001*
	*C. albicans*	0.69383	0.13012	0.000*
	*C. krusei*	1.56613	0.24545	0.000*
	*C. tropicalis*	1.21575	0.13515	0.000*
*C. tropicalis*	*C. glabrata*	-0.45442	0.14008	0.039*
	*C. albicans*	-0.52192	0.11451	0.002*
	*C. krusei*	0.35038	0.23755	0.829
	*C. parapsilosis*	-1.21575	0.13515	0.000*

*P < .05, SE: Standard Error

The result of Tamhane test showed a statistically significant difference between the adhesion amounts of *C. parapsilosis* and the other *Candida* species (*P* < .05), while no statistically significant difference was observed between *C. glabrata* and *C. albicans* (*P* = 1.000) and between *C. krusei* and *C. tropicalis* (*P* = .829).

The amount of *Candida* species adhering to materials produced at a 45-degree printing orientation was evaluated using Welch ANOVA (FWelch (4,27,167) = 58,528, *P* < .05) followed by a post hoc Tamhane test for further analysis. The results are presented in Table 6.

**Table 6:** The adhesion quantity of *Candida* species on materials fabricated with a 45-degree printing orientation (Tamhane test)

*Candida*		Mean Difference	SE	*P*
*C. glabrata*	*C. albicans*	-0.13856	0.09279	0.802
	*C. krusei*	1.47737	0.17843	0.000*
	*C. parapsilosis*	-0.59533	0.10456	0.000*
	*C. tropicalis*	0.69811	0.09145	0.000*
*C. albicans*	*C. glabrata*	0.13856	0.09279	0.802
	*C. krusei*	1.61593	0.17756	0.000*
	*C. parapsilosis*	-0.45677	0.10306	0.002*
	*C. tropicalis*	0.83667	0.08973	0.000*
*C. krusei*	*C. glabrata*	-1.47737	0.17843	0.000*
	*C. albicans*	-1.61593	0.17756	0.000*
	*C. parapsilosis*	-2.07270	0.18398	0.000*
	*C. tropicalis*	-0.77926	0.17686	0.006*
*C. parapsilosis*	*C. glabrata*	0.59533	0.10456	0.000*
	*C. albicans*	0.45677	0.10306	0.002*
	*C. krusei*	2.07270	0.18398	0.000*
	*C. tropicalis*	1.29344	0.10185	0.000*
*C. tropicalis*	*C. glabrata*	-0.69811	0.09145	0.000*
	*C. albicans*	-0.83667	0.08973	0.000*
	*C. krusei*	0.77926	0.17686	0.006*
	*C. parapsilosis*	-1.29344	0.10185	0.000*

*P < .05, SE: Standard Error

According to Tamhane test result; statistically significant differences were found among the *Candida* species (*P* < .05), except for *C. albicans* and *C. glabrata* (*P* = .802), which showed no significant difference.

The amount of *Candida* species adhering to materials produced at a 90-degree printing orientation was evaluated using ANOVA (F(4,59) = 41,131, *P* < .05), followed by a post hoc Tamhane test for further analysis. The results are presented in Table 7.

**Table 7:** The adhesion quantity of *Candida* species on materials fabricated with a 90-degree printing orientation (Tamhane test)

*Candida*		Mean Difference	Standard Error	*P*
*C. glabrata*	*C. albicans*	-0.20907	0.17581	1.000
	*C. krusei*	1.30541	0.17581	0.000*
	*C. parapsilosis*	-0.72961	0.17581	0.001*
	*C. tropicalis*	0.69994	0.17581	0.002*
*C. albicans*	*C. glabrata*	0.20907	0.17581	1.000
	*C. krusei*	1.51448	0.17581	0.000*
	*C. parapsilosis*	-0.52055	0.17581	0.045*
	*C. tropicalis*	0.90900	0.17581	0.000*
*C. krusei*	*C. glabrata*	-1.30541	0.17581	0.000*
	*C. albicans*	-1.51448	0.17581	0.000*
	*C. parapsilosis*	-2.03503	0.17581	0.000*
	*C. tropicalis*	-0.60548	0.17581	0.011*
*C. parapsilosis*	*C. glabrata*	0.72961	0.17581	0.001*
	*C. albicans*	0.52055	0.17581	0.045*
	*C. krusei*	2.03503	0.17581	0.000*
	*C. tropicalis*	1.42955	0.17581	0.000*
*C. tropicalis*	*C. glabrata*	-0.69994	0.17581	0.002*
	*C. albicans*	-0.90900	0.17581	0.000*
	*C. krusei*	0.60548	0.17581	0.011*
	*C. parapsilosis*	-1.42955	0.17581	0.000*

*P < .05. SE: Standard Error

According to Tamhane test result; statistically significant differences were found among the *Candida* species (*P* < .05), except for *C. albicans* and *C. glabrata* (*P* = 1.000), which showed no significant difference.

Based on the ANOVA test results, no statistically significant differences were observed in the adhesion of *C. glabrata* (F(2,23) = 0.828, *P* > .05), *C. albicans* (F (2,35) = 0.304, *P* > .05), *C. parapsilosis* (F(2,35) = 0.194, *P* > .05) to the materials made at different printing orientations. However, there is a statistically significant difference between the adhesion of *C. krusei* (F(2,35) = 5.802, *P* < .05) and *C. tropicalis* (F(2,35) = 8.430, *P* < .05) to materials produced at different angles. The Bonferroni test was used for pairwise comparisons. The Bonferroni test results for *C. krusei* are shown in Table 8, while those for *C. tropicalis* are shown in Table 9.

**Table 8:** Graph showing the amount of adhering *C. Krusei* (Bonferroni test)

Printing Orientation		Mean Difference	SE	*P*
0°	45°	0.71446^*^	0.23979	0.016*
	90°	0.70012^*^	0.23979	0.019*
45°	0°	-0.71446^*^	0.23979	0.016*
	90°	-0.01435	0.23979	1.000
90°	0°	-0.70012^*^	0.23979	0.019*
	45°	0.01435	0.23979	1.000

*P < .05, SE: Standard Error

**Table 9:** Graph showing the amount of adhering *C. Tropicalis* (Bonferroni test)

Printing Orientation		Mean Difference	SE	*P*
0°	45°	0.28558^*^	0.10982	0.041*
	90°	0.44501^*^	0.10982	0.001*
45°	0°	-0.28558^*^	0.10982	0.041*
	90°	0.15943	0.10982	0.468
90°	0°	-0.44501^*^	0.10982	0.001*
	45°	-0.15943	0.10982	0.468

*P < .05, SE: Standard Error

When considering the adhesion of *C. krusei* and *C. tropicalis*, there is a statistically significant difference between materials produced at 0-degrees of printing orientation compared to those produced at 45 and 90-degrees of printing orientation (*P* < .05). However, there is no statistically significant difference between materials produced at 45 and 90-degrees printing orientation (*P* > .05).

In this study, *C. parapsilosis* exhibited the greatest ability to adhere to surfaces across all tested printing orientations, as evidenced by the statistically significant higher adhesion values compared to other *Candida* species (*P* < .05). Specifically, *C. parapsilosis* showed significantly higher adhesion compared to *C. albicans*, *C. glabrata*, *C. krusei*, and *C. tropicalis* (Tables 4, 5 and 6).

**Fig. 2:**
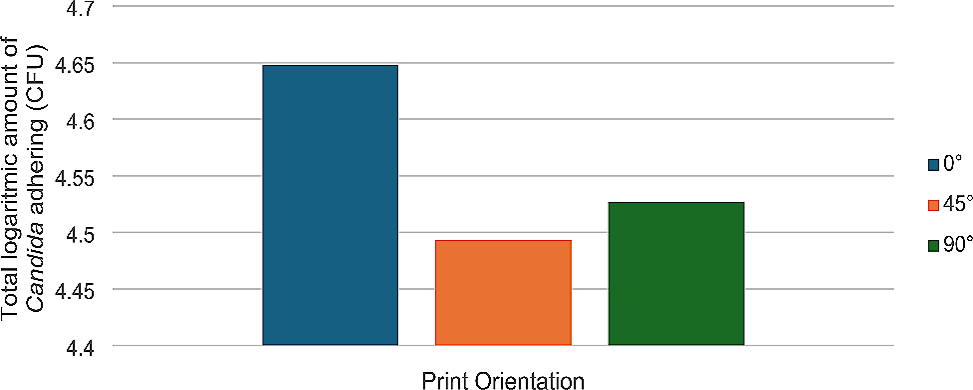
Total amount of *Candida* adhering to materials fabricated at different printing orientations

Descriptive statistics of the determination of the amount of *Candida* species adhesion to the materials by Real-Time PCR are given in Table 10; Fig. 2.

**Table 10:** Descriptive statistics for the amount of *Candida* species adhering to materials fabricated at different angles

Printing Orientation	*N*	Mean	SD	Minimum	Maximum
0°	12	4.6482	0.32941	4.10	5.21
45°	12	4.4936	0.20852	4.24	4.87
90°	12	4.4398	0.51896	3.29	5.23
Total	36	4.5272	0.37475	3.29	5.23

SD: Standard Deviation

According to the ANOVA test result, there is no statistically significant difference between the materials fabricated at different angles in terms of total *Candida* adherence according to Real-Time PCR analysis (F(2,23) = 1,000, *P* > .05).

The degree of adhesion of *Candida* strains was significantly affected by the surface roughness of the materials. Specifically, materials fabricated at a 45-degree printing orientation, which exhibited the highest surface roughness values (mean Ra = 3.4201 ± 1.3014 μm), showed significantly higher adhesion of *Candida* species compared to those fabricated at 0-degree and 90-degree orientations (Tables 2 and 3). The enhanced adhesion on rougher surfaces can be attributed to the increased surface area and potential for mechanical interlocking.

**Fig. 3:**
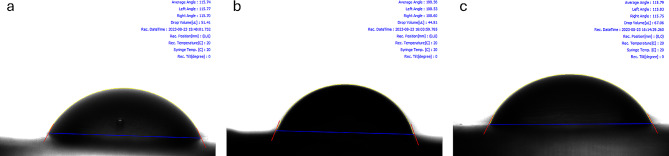
Contact angle images at different printing orientations **(a)** at 0-degree **(b)** at 45-degree **(c)** at 90-degree

Contact angle images are shown in Fig. 3.

It was found that the average contact angle value of the materials fabricated at a 45-degree printing orientation (111.16) was lower compared to those fabricated at 0 (116.79) and 90-degrees printing orientation (119.29).

**Fig. 4:**
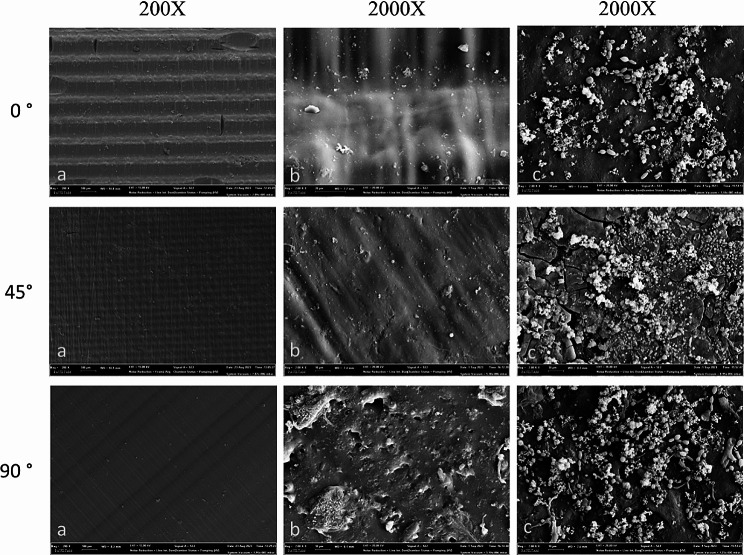
FESEM images of the material fabricated at 0, 45 and 90-degree orientation. **(a)** Control group at 200X **(b)** Vortexed material at 2000X **(c)** Vortexed material after adhesion at 2000X

One sample each from the control group, the vortexed material, and the vortexed material after adhesion were examined by FESEM at 200X and 2000X magnification. The FESEM images are shown in Fig. 4.

In the FESEM image of the 0-degree printing orientation material, horizontal surface lines resulting from the manufacturing angle can be seen. Both horizontal and vertical surface cracks were detected and are believed to be due to the manufacturing process. Although less yeast adhesion is observed in the vortexed samples compared to the non-vortexed samples in all groups, this result is expected.

In the FESEM image of the material with a 45-degree printing orientation, rectangular patterns formed by horizontal and vertical surface lines resulting from the printing orientation are visible. Deep cracks and defects can be seen in the non-treated sample. This may be due to the stepped manufacturing process associated with the 45-degree printing orientation.

In the FESEM image of the material with a 90-degree compression orientation, diagonal lines from the compression orientation are visible. Deep cracks and defects can be seen in the non-treated sample. This may be due to the stepped production process associated with the 45-degree printing orientation. Pores and rough areas on the surface were observed in the vortexed samples. This observation may be related to residual monomers that sometimes appear on the surface of 3D printed materials that have undergone post-processing. Although less yeast adhesion is observed in the vortexed samples compared to the non-vortexed samples, this result isexpected.

## Discussion

In this study, the adhesion of five different *Candida* species was investigated on disk-shaped materials produced by AM at three different printing angles. Statistically, the printing orientation has no effect on the adhesion of *Candida* species. However, there is a difference in the amount of adhesion between certain *Candida* species.

3D printing methods have been studied by numerous researchers [4, 24–26]. Andjela et al. [25] found that while SLA and DLP fabrication techniques are frequently investigated in research, there is a lack of studies on LCD fabrication techniques. In addition, while bar-shaped samples have been used for mechanical testing, disk-shaped samples have been used for microbial adhesion studies [4, 13, 20, 27–31]. Disk-shaped samples were fabricated according to ISO 10993-12:2021 standard with a diameter of 10 mm and a thickness of 2 mm at 0, 45, and 90-degree printing orientations, which is consistent with previous studies [24, 28, 31, 32].

Researchers [33–35] have demonstrated that *Candida* colonizes plastic surfaces. In this study, the resin material used in the manufacture of surgical guides, which aid in the precise placement of dental implants in their optimal positions, was used in procedures such as auto-transplantation, guided osteotomy, and root resection [23, 36].

Various results have been reported regarding the effect of printing orientation of materials on microbial adhesion [4, 24]. Shim et al. [4], performed a comparison of roughness and *C. albicans* adhesion levels of the materials they fabricated at 0, 45, and 90-degrees of printing orientation and found that the average roughness values of the specimens fabricated at 45-degree of printing orientation were higher compared to those at 0 and 45-degrees of print orientation. These results are consistent with those reported in our study. Shim et al. [4] also indicated that samples fabricated at a 45-degree printing orientation were rougher due to the gradual addition of layers, resulting in stepped edge surfaces between layers and increased surface roughness. The observation that samples produced at 45-degrees printing orientation had higher roughness values in this study may be attributed to this circumstance. The FESEM images from this study also support this finding. In addition, they found that *C. albicans* exhibited greater adhesion to materials fabricated at a 0-degree printing orientation compared to those fabricated at 45 and 90-degrees. Shim et al. [4] attributed this to the pore structure of the 0-degree printing orientation materials. In addition, Singh et al. [37] stated in their study that the porous structure of the material acts as a reservoir for microorganisms. In our study, although materials fabricated with a production orientation of 45-degree have higher average roughness values than those fabricated with 0 and 90-degrees, there was a noticeable preference for *C. krusei* and *C. tropicalis* to adhere to materials fabricated with a 0-degree orientation rather than those fabricated with 45 and 90-degrees. The results could be attributed to the explanations proposed by Shim et al. [4] and Singh et al. [37].

Li et al. [22], concluded that the printing orientation (0, 45 and 90-degrees) had no effect on the adhesion of *C. albicans* to the 3D printed denture bases. The same result can be found in our study with respect to *C. albicans*, *C. glabrata* and *C. parapsilosis*.

In a previous study, researchers found that *C. glabrata* showed twice as much adhesion to denture acrylic surfaces as *C. albicans* [38]. Contrary to this finding, in our study, there is no statistical difference between the adhesion levels of *C. glabrata* and *C. albicans* on the samples produced at all printing orientations. In another study investigating *Candida* adhesion to the silicone surface using Real-Time PCR analysis, *C. glabrata* was found to have statistically higher adhesion compared to *C. tropicalis* [39]. While these results are consistent with our study results, it is noteworthy that *C. glabrata* showed greater adhesion than *C. parapsilosis*, which is in contrast to our study. *C. parapsilosis* showed statistically higher adhesion than *C. glabrata* to the materials produced in all printed orientations in our study. On the contrary, Silva-Dias et al. [40] found that *C. parapsilosis* showed greater adhesion than *C. albicans*, which is in agreement with our study.

Luo and Samaranayake [38] emphasized the critical importance of examining a wide range of isolates within a single species before drawing conclusions about interspecies differences in phenotypic characteristics. Considering all these results, the difference between our results and previous studies may be attributed to the simultaneous evaluation of cells belonging to five *Candida* species.

Thaweboon et al. [41], investigated the adhesion of *C. albicans*, *C. glabrata*, *C. tropicalis*, and *C. krusei* to vanillin-incorporated PMMA resin for oral obturators. Statistically, *C. albicans* showed the highest amount of adhesion, followed by *C. tropicalis*, *C. glabrata*, and *C. krusei*, respectively. Although the results of the study are similar, the main difference is that there is no statistical difference between *C. glabrata* and *C. albicans* in our study.

He et al. [42], investigated the in vitro adhesion levels of *C. albicans, C. glabrata*, and *C. krusei* to prosthetic acrylic resin. They found that *C. glabrata* exhibited the highest level of adhesion. In this study, *C. glabrata* showed higher adhesion compared to *C. krusei*, while there was no significant difference between *C. glabrata* and *C. albicans*.

Studies [39, 43–46], have shown that the higher adhesion of one species compared to another is highly dependent on the substrate used and growth conditions. In addition, it is believed that the variation in the amount of adhesion may be due to different affinities and different cell wall compositions of the species and methodological variations of the studies [40]. The results of this study should be evaluated in light of all these factors.

When examining the FESEM images, we observed horizontal surface lines resulting from the printing orientation on materials produced at 0-degree; small squares formed by both horizontal and vertical surface lines due to the printing orientation on materials produced at 45-degree; and oblique lines resulting from the printing orientation on materials produced at 90-degree.

*Candida* adhesion is affected by variations in the surface geometry of materials. To mitigate yeast species adhesion and ensure effective cleanability, it is imperative to prevent the formation of porous structures. Compared to a more vertical printing orientation, there is a higher probability of unreacted monomer remaining in samples produced at a 0-degree printing orientation. *Candida* species showed greater adhesion to samples fabricated at a 45-degree angle compared to those produced at a 90-degree angle which is not statistically significant. Materials produced at a 45-degree printing orientation exhibit more irregularities compared to those produced at a 90-degree printing orientation. It was observed that *Candida* species tended to colonize these irregularities. Surface free energy is one of the most important factors in determining yeast adhesion to the intraoral device [47]. The surface properties of the material obtained with a 45-degree printing orientation that favor the adhesion of microorganisms, including lower hydrophobicity and higher surface energy, are likely the underlying reasons for the increased amount of *Candida* present on the surface. It is defined as the interaction between cohesion and adhesion forces and determines the wettability of the surface [47]. A linear relationship has been demonstrated between contact angle measurements and *Candida* adhesion on various materials. Increased surface free energy corresponds to increased microbial. Alternatively, as the surface becomes less hydrophobic, the presence of adherent microorganisms tends to increase [48–50].

The ability of microorganisms to adhere to oral devices has been linked to the chemical composition of the material, surface properties, or both [51]. Although the relationship between surface hydrophobicity and *Candida* adhesion has been extensively studied in the literature, data on this topic remain controversial [52–55]. Cerca et al. [51], reported in their study, aimed at analyzing the adhesion of various clinical *S. epidermidis* isolates to different materials, that greater biofilm formation was observed on hydrophilic surfaces compared to hydrophobic surfaces. A positively charged surface promotes greater microbial adhesion compared to a negatively charged surface [56]. In additionally, rough surfaces create an environment in which microorganisms are protected from brushing, muscle movement, and salivary flow, factors that can influence microbial adhesion [57].

Rapid and accurate identification of *Candida* species in clinical specimens is important for initial of appropriate antifungal therapy and patient care. Traditional culture methods for identification of *Candida* species are time-consuming and results can sometimes be inconclusive [58]. Recent clinical reports using advanced diagnostic methods based on molecular tools have identified abundant non-C. *albicans* (NCA) *Candida* species in primary and recurrent infections [22, 58]. *C. glabrata*, *C. tropicalis*, *C. krusei*, and *C. parapsilosis* have been isolated from patients with oropharyngeal candidiasis, including those with removable dentures, HIV infection, diabetes, or other oral complications [59]. Many studies have used Real Time-PCR as a better alternative to conventional PCR [60–65].

Our findings demonstrate that *C. parapsilosis* has a significantly higher ability to adhere to surfaces compared to other *Candida* species. This is consistent with previous research indicating the robust adhesion properties of *C. parapsilosis*, particularly in medical and dental contexts [40]. Additionally, the impact of surface roughness on *Candida* adhesion was clearly evident, with rougher surfaces facilitating greater microbial adherence. This aligns with existing literature suggesting that increased surface roughness enhances microbial retention due to larger surface area and mechanical interlocking opportunities.

## Conclusion

This study investigated the adhesion abilities of different *Candida* species to various surfaces. Our findings revealed that *C. parapsilosis* exhibited the greatest adhesion capability compared to other *Candida* species. Additionally, surface roughness significantly influenced the degree of *Candida* adhesion, with rougher surfaces showing higher adhesion rates. These results provide crucial insights for the design and material selection of dental and medical devices.

This study contributes valuable data to the microbiology community, particularly in understanding the adhesion mechanisms of *Candida* species. The findings can inform new strategies for preventing and controlling *Candida* infections. Moreover, a deeper understanding of the biofilm-forming potential of these species can lead to novel approaches in clinical practice for managing and treating infections. Future research should aim to validate these findings in clinical settings and explore the effects of surface modifications on infection control.

## Data Availability

The datasets used and/or analysed during the current study available from the corresponding author on reasonable request.
